# Disruption of Tip60 HAT mediated neural histone acetylation homeostasis is an early common event in neurodegenerative diseases

**DOI:** 10.1038/s41598-020-75035-3

**Published:** 2020-10-26

**Authors:** Mariah Beaver, Akanksha Bhatnagar, Priyalakshmi Panikker, Haolin Zhang, Renee Snook, Visha Parmar, Gayathri Vijayakumar, Niteesha Betini, Sunya Akhter, Felice Elefant

**Affiliations:** Department of Biology, Drexel University, 3245 Chestnut Street, PISB 312, Philadelphia, PA 19104 USA

**Keywords:** Neuroscience, Diseases of the nervous system, Epigenetics in the nervous system, Learning and memory, Synaptic plasticity

## Abstract

Epigenetic dysregulation is a common mechanism shared by molecularly and clinically heterogenous neurodegenerative diseases (NDs). Histone acetylation homeostasis, maintained by the antagonistic activity of histone acetyltransferases (HATs) and histone deacetylases (HDACs), is necessary for appropriate gene expression and neuronal function. Disruption of neural acetylation homeostasis has been implicated in multiple types of NDs including Alzheimer’s disease (AD), yet mechanisms underlying alterations remain unclear. We show that like AD, disruption of Tip60 HAT/HDAC2 balance with concomitant epigenetic repression of common Tip60 target neuroplasticity genes occurs early in multiple types of *Drosophila* ND models such as Parkinson’s Disease (PD), Huntington’s Disease (HD) and Amyotrophic Lateral Sclerosis (ALS). Repressed neuroplasticity genes show reduced enrichment of Tip60 and epigentic acetylation signatures at all gene loci examined with certain genes showing inappropriate HDAC2 repressor enrichment. Functional neuronal consequences for these disease conditions are reminiscent of human pathology and include locomotion, synapse morphology, and short-term memory deficits. Increasing Tip60 HAT levels specifically in the mushroom body learning and memory center in the *Drosophila* brain protects against locomotion and short-term memory function deficits in multiple NDs. Together, our results support a model by which Tip60 protects against neurological impairments in different NDs via similar modes of action.

## Introduction

Neurodegenerative diseases (NDs) are a heterogeneous group of disorders marked by progressive loss of neuronal cells in the brain and/or the spinal cord. Some common NDs include Alzheimer’s Disease (AD), Huntington’s. Depending on the specific subset of neurons and/or the area of nervous system severely affected, NDs vary with respect to the molecular pathways underlying the disease and the subsequent clinical manifestations^1^. Despite exhibiting a wide range of molecular and phenotypic heterogeneity, NDs share some general characteristics, such as protein misfolding and aggregation, mitochondrial dysfunction, dysregulation of similar molecular/cellular pathways and loss of chromatin dynamics^2–4^. Together, these similarities support convergence of heterogeneous NDs, and better insight into these common links will help target multiple NDs with potential unifying therapeutics^3,4^.

Over the past decade, histone acetylation mediated epigenetic mechanisms have been implicated in a variety of neural processes required for brain function that include neuronal differentiation and maintenance of activity-dependent synaptic plasticity in mature neurons^5,6^. Acetylation homeostasis in the brain is maintained by the antagonistic activity of histone acetyltransferases (HATs) and histone deacetylases (HDACs), and is essential for proper neuronal gene expression and concomitant neural health^4,7^. Compelling evidence demonstrates that histone acetylation dysregulation contributes to age related cognitive impairment and age associated neurodegenerative diseases, such as HD, PD, ALS and AD^5,7–10^. For instance, the polyglutamate repeat region in HD was shown to sequester HATs CREB-binding protein (CBP) and p300/CBP associated factor (PCAF), resulting in global reduction of H3 and H4 acetylation levels and altered gene expression^11^. In PD, associated α-synuclein can inhibit H3 histone acetylation by CBP, p300 and PCAF, causing apoptosis in human neuroblastoma cells^12^ and mitochondrial dysfunction^13^. In a mouse model of ALS, loss of CBP HAT activity was observed^14^. Additionally, impaired expression levels of class I, II and IV HDACs and Sirtuins have been observed in human postmortem ALS brain and spinal cord^15,16^. In line with these studies, we previously reported decreased levels of the HAT, Tat interactive protein 60 kDa (Tip60), and concomitant loss of Tip60 associated specific histone acetylation marks in an AD *Drosophila* model and in human postmortem AD brain. We further demonstrated that increasing Tip60 levels in the AD *Drosophila* brain protects against alteration of Tip60 HAT/HDAC2 balance, epigenetic-mediated neuroplasticity gene repression and functional cognitive deficits that occur during early stages of neurodegenerative progression^17^. These findings raise the possibility that Tip60 HAT/HDAC2 imbalance may be an early common event that contributes to epigenetic mediated gene misregulation and cognition deficits in other NDs as well.

Here, we test the hypothesis that Tip60 mediated histone acetylation homeostasis is disrupted during early stages of neurodegeneration in multiple types of NDs. We show that disruption of Tip60 HAT/HDAC2 balance with concomitant epigenetic repression of common neuroplasticity Tip60 target genes is an early event in multiple types of *Drosophila* ND models that include Parkinson’s Disease (PD), Huntington’s Disease (HD) and Amyotrophic Lateral Sclerosis (ALS). Functional neuronal consequences of each of these disease conditions are consistent with human pathology and include defects in locomotion, synapse morphology, and short-term memory deficits. Increasing Tip60 HAT levels in the mushroom body learning and memory center in the *Drosophila* brain protects against these neural deficits. Our findings are the first to demonstrate that disruption of Tip60 HAT/HDAC2 homeostasis and concomitant Tip60 gene control is a critical initial mechanism common to multiple neurodegenerative disorders.

## Results

### Common Tip60 target neuroplasticity genes are repressed during early stages of multiple types of neurodegenerative disorders

**Figure 1:**
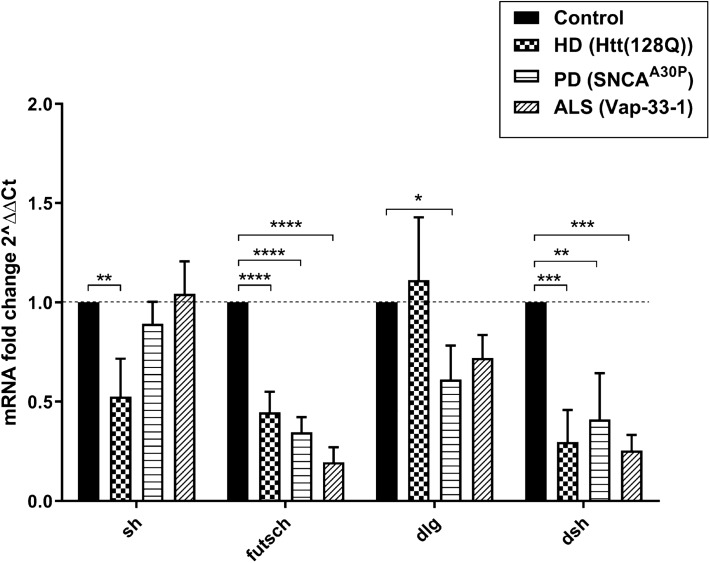
Common synaptic plasticity genes are repressed during early stages of neurodegeneration in HD, PD and ALS *Drosophila* models. Real-time PCR was performed using 40 staged third instar larval brain tissue from larvae expressing Htt(128Q), SNCA^A30P^ or Vap-33-1 under elav^C155^ pan-neuronal Gal4 driver. Control depicts elav^C155^ Gal4 driver alone that is identical to respective UAS drivers alone for ALS, PD or Htt lines. Histogram represents relative fold change in gene expression relative to control flies. Data is from 3 independent experiments per genotype. Fold change was calculated using ∆∆Ct method using the internal *Drosophila* RP49 reference gene as control. Statistical significance was calculated using one-way ANOVA with Dunnett’s multiple comparison test to compare w^1118^ to each of the other genotypes. *p < 0.05, **p < 0.01, ***p < 0.001, **** < 0.0001. Error bars represent SEM. (See supplementary Table S2 for primer sequences).

Transcriptional misregulation of critical neuronal genes in the brain are central late stage features of most neurodegenerative disorders including AD^18^, PD^19^, HD^20^ and ALS^21^. However, whether these gene expression alterations occur during early stages of disease progression remains to be elucidated. Our previous transcriptional analysis^17^ suggested that Tip60-mediated neuronal transcriptional dysregulation is an early event in AD progression. To test whether this is a common early event in additional types of NDs, we assessed expression of four Tip60 neuroplasticity genes using RNA isolated from the brains of three distinct and well-characterized early-stage *Drosophila* ND third instar larval models: the HD associated model expressing 128 poly-Q repeats in the human Huntingtin (Htt) gene (Htt(128Q))^22^, PD associated model expressing mutated form of human α-synuclein (SNCA^A30P^)^23^ and ALS associated model overexpressing Vap-33-1, a *Drosophila* homolog of VAMP-associated protein (Vap-33-1)^24^. These disease genes were inducibly and specifically expressed in all neurons using the pan-neuronal *elav-Gal4* driver. The four synaptic plasticity genes, *shaker (sh)*, *futsch, discs large (dlg)* and *dishevelled (dsh),* were selected because they are conserved within mammals, are *bona fide* direct targets of Tip60 HAT and their expression was repressed during early stages of APP mediated neurodegeneration and restored by increasing Tip60 in the brain^17^. We observed that *futsch*, a microtubule-associated protein essential in promoting synaptic bouton formation ^25^, and *dsh*, a key component of the Wnt- signaling pathway^26^ were repressed in the brain during early stages of all three neurodegenerative conditions (Fig. 1: F(3, 8) = 65.32, p < 0.0001; and F(3, 8) = 16.59, P = 0.0009, one-way ANOVA with Dunnett’s multiple comparison test). Additionally, *sh*, an ion channel involved in activity dependent synaptic plasticity^27^ was repressed in HD disease progression (Fig. 1: F(3, 8) = 8.785, p = 0.0065, one-way ANOVA with Dunnett’s multiple comparison test). Moreover, *dlg*, a scaffold protein essential for proper synapse formation^28^, was repressed in PD neurodegeneration (Fig. 1: F(3, 8) = 4.608, p = 0.0373, one-way ANOVA with Dunnett’s multiple comparison test). Together, these findings suggest that transcriptional dysregulation of certain synaptic plasticity genes is an early onset event common to multiple types of neurodegenerative disorders.

### Disruption of Tip60 HAT/ HDAC2 expression levels is an early event common to multiple types of neurodegenerative disease models

**Figure 2:**
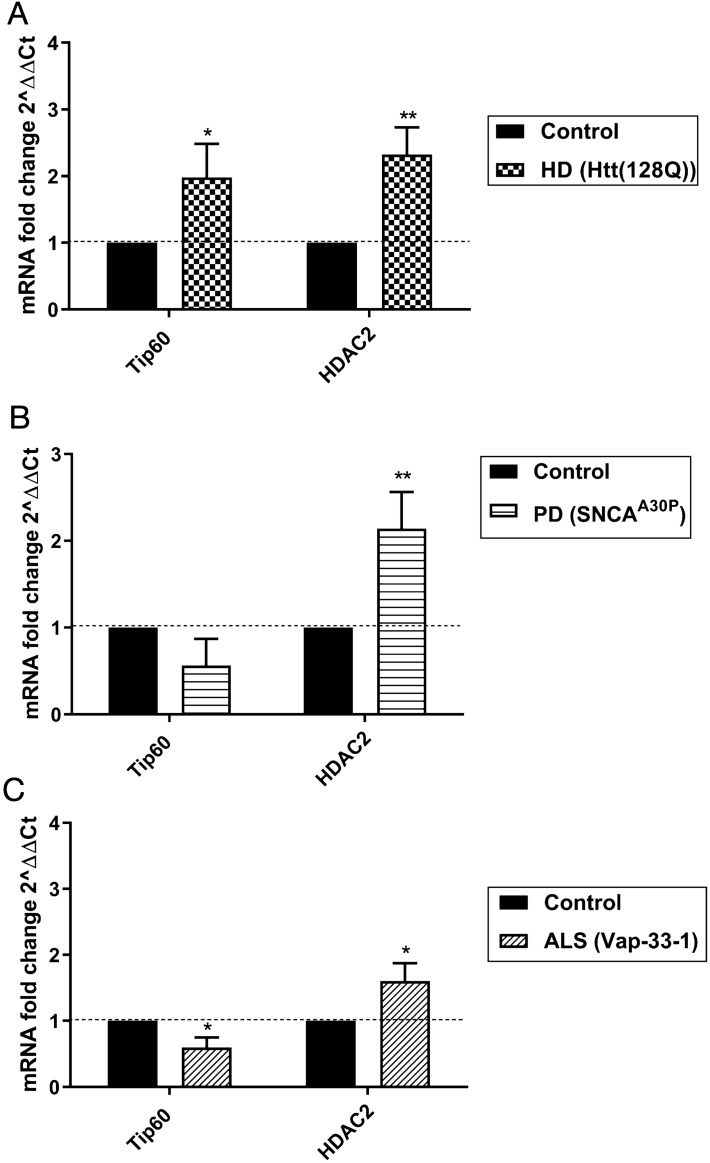
Disruption of Tip60/HDAC2 transcriptional levels is a common early event in multiple neurodegenerative disorders. Real-time PCR was performed using 40 staged third instar larval brain tissue from larvae expressing **(A)** Htt(128Q), **(B)** SNCA^A30P^ or **(C)** Vap-33-1 under elav^C155^ pan-neuronal Gal4 driver. Control depicts elav^C155^ Gal4 driver alone that is identical to respective UAS drivers alone for ALS, PD or Htt lines. Histogram represents relative fold change in gene expression relative to control flies. Data is from 3 independent experiments per genotype. Fold change was calculated using ∆∆Ct method using the internal *Drosophila* RP49 reference gene as control. Statistical significance was calculated using unpaired Student’s t-test. *p < 0.05, **p < 0.01. Error bars represent SEM. (See Appendix A for primer sequences).

We previously demonstrated that Tip60 HAT/HDAC2 balance is disrupted in the AD larval brain during the early stages of APP mediated neurodegenerative progression. To test whether disruption of Tip60 HAT/HDAC2 balance is a common early stage feature in additional NDs, we assessed expression levels of Tip60, and *Drosophila* HDAC1/2 ortholog, Rpd3, using RNA isolated from larval brains that model early neurodegenerative progression in each of the PD, HD and ALS conditions. As HDAC2 and Rpd3 play conserved roles in memory formation and synaptic activity in mouse and *Drosophila*, respectively^29–31^ we refer to Rpd3 here as an HDAC2 homolog**.** We observed a significant increase in *HDAC2* (*Rpd3*) mRNA levels in HD, PD and ALS *Drosophila* larval brain (Fig. 2A: (t(4) = 5.675, p = 0.0048, unpaired t-test); Fig. 2B: (t(4) = 4.676, p = 0.0095, unpaired t-test); Fig. 2C: (t(4) = 3.904, p = 0.0175, unpaired t-test) in comparison to wild-type control brains. In contrast, *Tip60* showed slight but non-significant reduction in the PD *Drosophila* brain (Fig. 2B: (t(4) = 2.468, p = 0.0691, unpaired t-test), significantly reduced in the ALS *Drosophila* brain (Fig. 2C: (t(4) = 4.589, p = 0.0101, unpaired t-test) and upregulated in the HD *Drosophila* brain (Fig. 2A:t(4) = 3.392, p = 0.0275, unpaired t-test).

**Figure 3:**
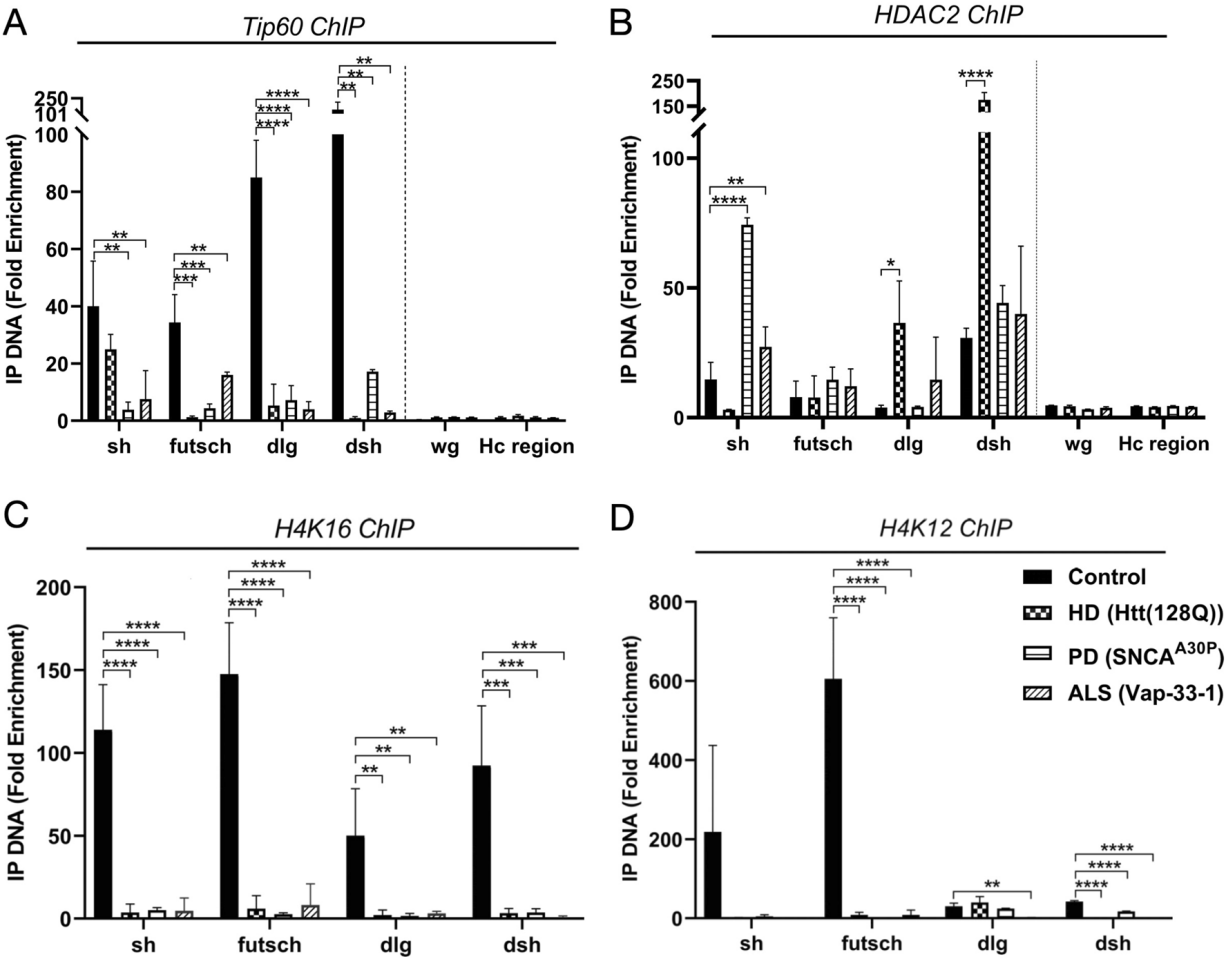
Alteration of Tip60/HDAC2 binding patterns and reduction of histone acetylation is an early event in multiple neurodegenerative disorders. Chromatin was isolated from 100 pooled larval heads for each indicated genotype under the elav^C155^pan-neuronal Gal4 driver. Control depicts elav^C155^ Gal4 driver alone that is identical to respective UAS drivers alone for ALS, PD or Htt lines. **(A–D)**. Histogram representing ChIP enrichment using the **(A)** Tip60, **(B)** HDAC2, **(C)** H4K16Ac, **(D)** H4K12Ac antibodies. All data are from three independent experiments per genotype. Fold change shown is relative to the non-specific IgG antibody control. Negative specificity control primers that amplify a heterochromatin (hc) region within *Drosophila* chromosome 3L and primers for non-target gene specificity gene are depicted as hc and wg. Statistical significance was calculated using one-way ANOVA with Dunnett’s multiple comparisons. *p < 0.05, **p < 0.01, ***p < 0.001, ****p < 0.0001. Error bars indicate SEM.

We previously uncovered a mechanism underlying AD associated neuroplasticity gene repression in the brain involving inappropriate HDAC2 enrichment at specific Tip60 neuroplasticity target genes (*sh, dlg, futsch,* and *dsh*) during early stages of APP mediated neurodegeneration. To test whether similar mechanisms underly the Tip60 neuroplasticity gene repression we observed in the brains of PD, HD and ALS *Drosophila* models, we carried out ChIP-qPCR using Abs to Tip60 and HDAC2 to assess their recruitment at these same genes the PD, HD, and ALS larval heads. Strikingly, and similar to AD, enrichment of Tip60 binding was significantly reduced at *sh, futsch, dlg* and *dsh* gene loci in the larval brains of all three PD, HD, and ALS fly models (Fig. 3). Conversely, while we observed a significant increase in inappropriate HDAC2 enrichment at *sh* in both ALS and HD brains and *dlg* and *dsh* in HD larval brains, no additional inappropriate HDAC2 enrichment at gene loci was observed in any of the disease models. No enrichment was observed in wingless (wg) gene loci or repressed heterochromatin region (hc) specificity controls. We next used ChIP to assess acetylation enrichment at four repressed synaptic plasticity gene loci (*sh, dlg, futsch, dsh*) in PD, HD and ALS larval brains using Abs to well characterized Tip60-mediated cognition linked histone acetylated (Ac) marks AcH4K12 and AcH4K16. Similar to our previous findings in the fly AD model, we found reduced levels of AcH4K16 at all genes in all disease models, and reduced levels of AcH4K12 at all gene loci in all disease models except at *sh*. Together, these results suggest that disruption of Tip60 HAT homeostasis and concomitant epigenetic mediated neuroplasticity gene repression we previously observed in the AD brain is also an early common feature in PD, HD and ALS.

### Drosophila larvae modelling PD and ALS neurodegenerative conditions exhibit synaptic morphological alterations

Thus far, our findings demonstrated that repression of Tip60 neuroplasticity genes mediated by disruption of Tip60 HAT/HDAC2 balance is an early event in HD, PD, and ALS. Thus, we asked whether synaptic plasticity was negatively affected in these *Drosophila* models of NDs. The *Drosophila* NMJ is a highly dynamic structure that constantly changes in response to activity and body size, making it a powerful model to investigate synaptic plasticity^32,33^. Importantly, the NMJ provides relevance for understanding brain function as it shares central features with major excitatory synapses of the mammalian brain including ionotropic glutamate receptors and a variety of additional proteins also found in mammalian central synapses^32,34,35^.

**Figure 4:**
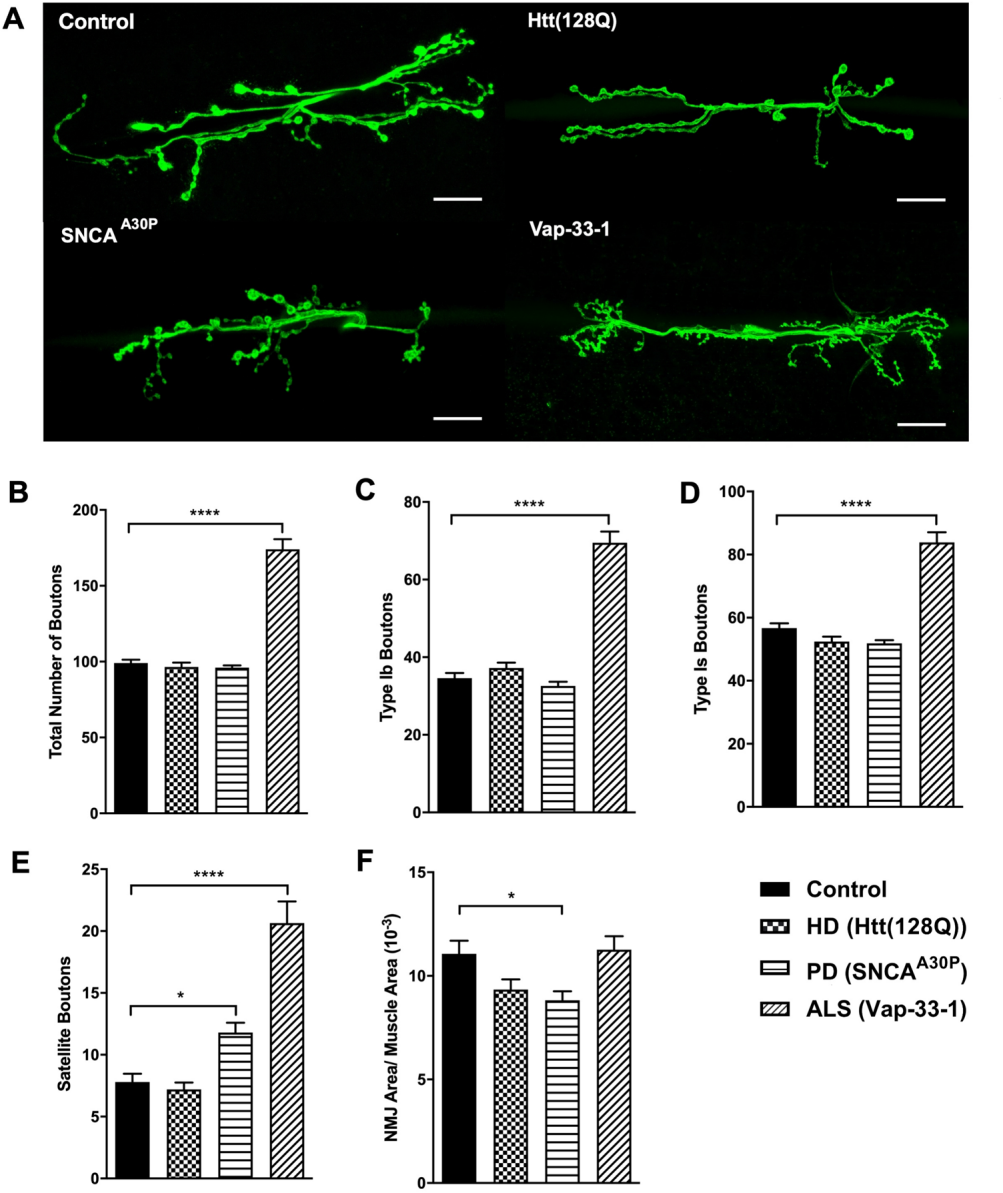
Synaptic abnormalities within the neuromuscular junction are present during early stages of neurodegeneration in HD, PD and ALS *Drosophila* models. NMJ dissections using staged third instar larvae expressing Htt(128Q), SNCA^A30P^ or Vap-33-1 under elav^C155^ pan-neuronal Gal4 driver. Control depicts elav^C155^ Gal4 driver alone that is identical to respective UAS drivers alone for ALS, PD or Htt lines **(A)** Confocal imaging analysis of larval NMJ of the indicated genotypes observed between muscles 6/7 of body segment A3. Presynaptic neuronal membrane stained by anti-HRP antibody staining (green). Scale bar represents 20 μm. **(B-F)** Quantitative analysis of the synaptic bouton morphology using NMJ images acquired through antibody staining. For each genotype, **(B)** the total number of boutons, **(C)**Type-Ib boutons, **(D)**Type-Is boutons and **(E)** satellite boutons were analyzed. The expansion of NMJ in each genotype is represented by **(F)** area of NMJ normalized to the muscle area. Each genotype is represented by 20 samples. Statistical significance between disease genotype versus control was calculated using one-way ANOVA with Dunnett’s multiple comparisons. Asterisks (*) indicate statistical significance with respect to wild type. *p ≤ 0.05, **p ≤ 0.01, ***p ≤ 0.001 and ****p ≤ 0.0001. All error bars depict SEM.

The NMJ present between muscles 6/7 of segment A3, containing glutamatergic Type-I synaptic boutons, has been extensively studied due to its resemblance with mammalian central synapses^34,35^. Thus, we explored whether there were alterations in synaptic morphology within this NMJ area in Htt(128Q), SNCA^A30P^ or Vap-33-1 expressed pan-neuronally with *elav-Gal4* driver (Fig. 4A). In comparison to the wild type control (*elav/*+), we observed a significant increase in the total number of boutons per NMJ in our ALS model (Fig. 4B: F(3, 76) = 98.75, p < 0.0001, one way ANOVA with Dunnett’s multiple comparison test). Type-I synaptic boutons can be further classified as Type-Ib (big) and Type-Is (small), which have an average diameter of 3–5 μm and 1–1.5 μm, respectively^36^. In addition to the bouton size, these boutons differ with respect to their distribution, mitochondrial abundance, vesicle density and composition, synaptic plasticity and ultimately show varying neuronal excitability^36,37^. Satellite boutons, that form via budding of small boutons from a parent bouton, can also be observed^35^. Using this classification, synaptic boutons were categorized as Type-Ib, Type-Is or satellite boutons (Fig. 4C-E). Type-Ib and Type-Is boutons were significantly upregulated in our ALS model (Fig. 4C: F(3, 76) = 92.96, p < 0.0001; and Fig. 4D: F(3, 76) = 57.63, p < 0.0001, one way ANOVA with Dunnett’s multiple comparison test). We also observed significant increase in satellite boutons in our PD and ALS models (Fig. 4E: F(3, 76) = 34.70, p < 0.0001, one way ANOVA with Dunnett’s multiple comparison test).

Since the area of contact between neurons and muscles is generally proportional to the level of neurotransmission^38^, we observed if there were differences in the area covered by NMJ. We observed a significant decrease in the area of NMJ relative to the muscle area in PD neurodegenerative conditions (Fig. 4F: F(3, 76) = 4.765, p = 0.0043, one way ANOVA with Dunnett’s multiple comparison test). Interestingly, the drastic increase in bouton number in the ALS model did not increase total NMJ area. This may be due to the relatively smaller size of boutons (Fig. 4A) and previously reported in literature^39^. Lastly, there was no change in the number of branches per NMJ per genotype (data not shown). Together, these results reveal that synaptic morphological defects are a central common feature during early neurodegenerative progression in the *Drosophila* models of PD and ALS and are consistent with the early alterations in epigenetic mediated synaptic plasticity gene expression we observe.

### Tip60 HAT action restores locomotor function in PD, HD, and ALS larvae

**Figure 5:**
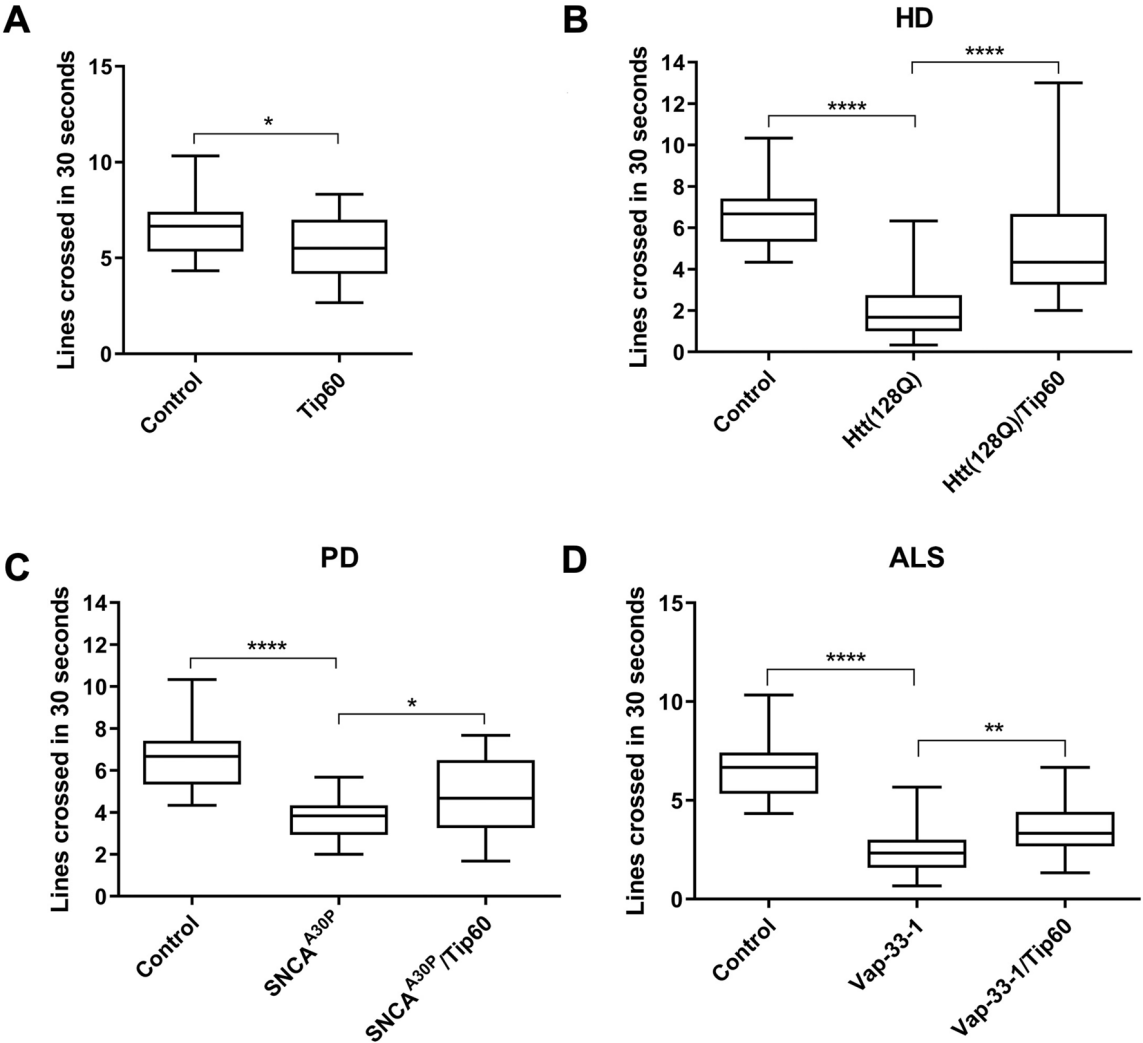
Tip60 mediated rescue of locomotion function in HD, PD and ALS *Drosophila* models. Locomotion assay using staged third instar larvae expressing Htt(128Q), SNCA^A30P^ or Vap-33-1 under the control of mushroom body (MB)-specific 201Y-Gal4 driver. Control depicts representative figure of 201Y-Gal4 driver alone that is identical to respective UAS drivers alone for ALS, PD or Htt lines. Line crossing assay was performed, where the number of lines crossed in 30 s by the head of a larvae on a grid was recorded. This assay was performed using 3rd instar larvae (n = 30) to compare locomotion function between (**A**) Control versus Tip60 induction alone using the 201Y-Gal4 driver; (**B**) Htt(128Q) and Htt(128Q)/Tip60; (**C**) SNCA^A30P^ and SNCA^A30P^/Tip60; and (**D**) Vap-33-1 and Vap-33-1/Tip60 under 201Y MB-specific Gal4 driver. For (A), statistical significance was calculated using unpaired Student’s t-test. One-way ANOVA with Tukey’s post hoc analysis was used to determine statistical significance between different genotypes indicated in (**B**–**D**). *p < 0.05, **p < 0.01, ****p < 0.0001. Error bars represent SEM.

Proper regulation of synapse activity is required for optimal functioning of the NMJ which in turn influences locomotor ability^40^. Our observation that NMJ associated synaptic morphological alterations were identified in all three NDs tested prompted us to ask whether concomitant locomotor deficits would be apparent as well. While early stage locomotor deficits in the HD fly model have been previously reported^41^, whether such deficits occur in PD and ALS fly models in this study were unknown. To assess whether locomotor function during early neurodegeneration is compromised, we performed a well characterized *Drosophila* larval line crossing assay, where the number of lines crossed in 30 s by the head of a larva placed on a grid were recorded. This assay was performed using third instar larvae expressing either Htt(128Q), SNCA^A30P^ or Vap-33-1 under the control of mushroom body (MB)-specific 201Y-Gal4 driver. This region in the *Drosophila* brain is associated with a diverse set of behavioral functions, including olfactory learning, short-term memory and locomotion^42,43^. Our results showed that Htt(128Q), SNCA^A30P^ and Vap-33-1 larvae were significantly less mobile and crossed significantly lesser grid lines when compared to control wild-type larvae (Fig. 5B–D). Together, these results indicate that early defects in locomotor function are a common feature in multiple neurodegenerative conditions.

Our lab has previously demonstrated that gene control is a key mechanism by which Tip60 exerts its epigenetic action in restoring function of a variety of neuronal processes^44^. To this end, we showed that increasing neuronal Tip60 levels in an AD associated *Drosophila* model restored locomotion deficits and concomitant expression of genes required for this function. To test whether increasing Tip60 levels would also rescue the locomotor deficits observed in HD, PD and ALS *Drosophila* models, we generated a *201Y-Gal4; UAS-Tip60* (201Y; Tip60) model system that enables us to modulate Tip60 HAT levels specifically in the MB, in vivo. We then performed the *Drosophila* line crossing locomotor assay using third instar larvae co-expressing both Htt(128Q), SNCA^A30P^ or Vap-33-1 along with increased levels of Tip60 in the MB. We observed a significant restoration of locomotor ability in HD, PD and ALS *Drosophila* larvae with increased Tip60 MB levels (Fig. 5B: F(2,87) = 37.87, p = 0.0001, one-way ANOVA with Tukey’s post hoc analysis; 5C: F(2,87) = 30.20, p = 0.0001, one-way ANOVA with Tukey’s post hoc analysis, 5D: F(2,87) = 82.05, p = 0.0001, one-way ANOVA with Tukey’s post hoc analysis). Increasing Tip60 levels in a wild-type background also showed defects in locomotor ability (Fig. 5A) suggesting that locomotor function likely requires appropriate levels of Tip60 mediated neuronal acetylation homeostasis. Together, these findings indicate that increasing Tip60 in the MB region of the larval brain in HD, PD and ALS fly models protect against early stage locomotion deficits in these multiple types of NDs.

### Tip60 HAT action improves short-term memory deficits in PD larvae

**Figure 6:**
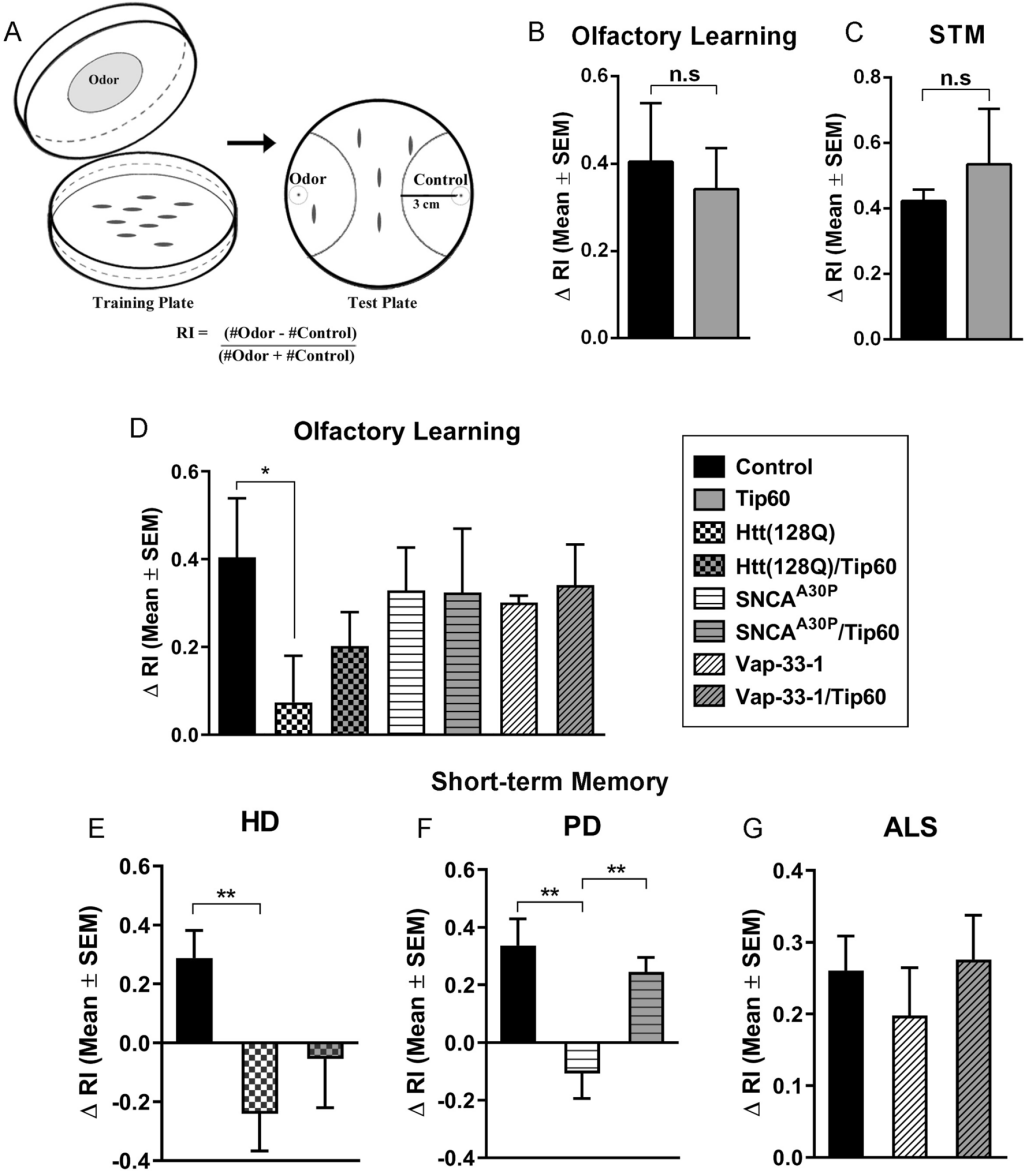
Defects in short-term memory in the PD *Drosophila* model is partially restored by increasing Tip60 HAT activity. Olfactory assay using staged third instar larvae expressing Htt(128Q), SNCA^A30P^ or Vap-33-1 under MB-specific 201Y-Gal4 driver. Control depicts representative figure of 201Y-Gal4 driver alone that is identical to respective UAS drivers alone for ALS, PD or Htt lines. **(A)** Olfactory training and test plates for larval associative learning. For training, third instar larvae were placed on 2.5% agar plates with a thin layer for 1 M SUC or DW with odorant (LIN) spotted on a filter disk on the lid. After 30 min of training, 50–100 larvae were transferred to the test plate. After 3 min, the number of larvae that moved in the semi-circular area was counted to determine the responsive index (RI). Olfactory learning and short-term memory performance for indicated genotypes plotted in ∆RI. ∆RI was calculated as the difference in RI of LIN/SUC and control LIN/DW (n = 150). Comparisons of larval olfactory associative **(B)** learning and **(C)** short-term memory between control and Tip60 induction alone using MB-specific *201Y-Gal4* driver indicating no significant difference between the two. Statistical significance for the two-way comparison between the control fly lines. Control versus Tip60 was calculated using unpaired Student’s t-test. **(D)** ∆RI indicating larval olfactory associative learning for the indicated genotypes under 201Y MB-specific Gal4 driver. Statistical significance was calculated using one- way ANOVA with Dunnett’s multiple comparisons test. Short-term memory performance indicated by larval olfactory response 30minutes after training for **(E)** HD, **(F)** PD and **(G)** ALS, plotted in ∆RI. For multiple comparisons test, one-way ANOVA with Tukey’s post hoc analysis was used to determine statistical significance between the three different genotypes indicated in (**E**–**G**). RIs of all the genotype were normalized using their respective locomotion speed for all experiments. *p < 0.05, **p < 0.01. Error bars represent SEM.

Our previous findings demonstrated that increasing Tip60 HAT activity partially suppresses learning and memory associated defects in the AD *Drosophila* larval and adult brain^17,45^. However, whether such cognitive deficits also occur early in other neurodegenerative disorders and can be prevented by increasing Tip60 HAT levels in the brain remain to be elucidated. To address these questions, we carried out single odor paradigm for olfactory associative learning and memory^46^, to assess the larval short-term memory (STM) using published methodology ^46^. Larvae expressing either Htt(128Q), SNCA^A30P^ or Vap-33-1 under the control of MB-specific *201Y-Gal4* driver were first conditioned to associate the given odor, linalool (LIN), to an appetitive gustatory reinforcer, sucrose (SUC). These larvae were exposed to LIN for 30 min on agar plate containing SUC. After conditioning, the larvae were tested for olfactory response on the test plate (Fig. 6A). Importantly, the higher response index (RIs) of all the genotype were normalized using their respective locomotion speed which is presented in Fig. 5. We compared the larval response after 0 min and 30 min of LIN/SUC conditioning, to test learning and STM, respectively. In comparison to the control larvae that had been exposed to LIN in conjunction with distilled water (DW), LIN/SUC conditioned control larvae and larvae expressing Tip60 induction under the MB-specific *201Y-Gal4* driver showed enhanced migration, as well as high ΔRI at 0 min (Fig. 6B: t(4) = 0.6606, p = 0.5449, unpaired t-test) and 30mins (Fig. 6C: t(8) = 1.449, p = 0.184, unpaired t-test), indicating normal learning and STM. However, the HD larvae showed significantly lower ΔRI at both 0 min (Fig. 6D: F(6,14) = 3.397, p = 0.0278, one-way ANOVA with Dunnett’s multiple comparisons test) and 30 min (Fig. 6E: F(2,6) = 14.15, p = 0.0054, one-way ANOVA with Tukey’s post hoc analysis) indicating defects in both learning and STM. Although PD larvae showed only slightly lower learning ability (Fig. 6D), significantly lower STM was observed in PD as compared to control w^1118^ larvae (Fig. 6F: F(2,6) = 26.63, p = 0.0010, one-way ANOVA with Tukey’s post hoc analysis), indicating significant defect in STM but not learning. Lastly, we observed no defects in learning and a non-significant trend in decreased STM ability in ALS larvae (Fig. 6D,G).

To test whether increasing Tip60 HAT activity could rescue learning and STM defects observed in HD and PD and ALS *Drosophila* larvae, we crossed fly strains expressing either Htt(128Q), SNCA^A30P^ or Vap-33-1 with *201Y;Tip60* for co-expression of disease-associated proteins and Tip60 in the mushroom body of the progeny. Of note, olfactory and gustatory responses of all genotypes were normal when compared to control 201Y-GAL4 driver alone (Supplementary Table 1). Increasing Tip60 HAT activity partially restored STM in the PD (Fig. 6F: F(2,6) = 26.63, p = 0.0010, one-way ANOVA with Tukey’s post hoc analysis) while there was a non-significant trend in rescue in HD larvae (Fig. 6D) and in ALS larvae (Fig. 6G). We also carried out this assay at multiple different time points during day and night, and in different locations with no significant variation in the results reported. Together these results reveal that an increase in Tip60 levels in the nervous system partially protects against early stage STM in PD and displays a non-significant trend towards protection in both ALS and HD *Drosophila* larvae.

## Discussion

Loss of global histone acetylation and concomitant alteration of neural gene expression profiles has been reported in animal models of different NDs that include AD, PD, HD and ALS^4,14,47–49^. Nevertheless, how global histone hypoacetylation and concomitant chromatin dysregulation occurs in the nervous system in these different NDs remains poorly understood. Further, the molecular heterogeneity of NDs adds an additional level of complexity, making it unclear whether a common epigenetic mechanism underlies the neural acetylation disruption that accompanies multiple NDs^4,7^. Here we use a variety of epigenetic, morphological and behavioral assays to assess convergent and divergent features between PD, HD and ALS which is summarized in Supplemental Table 3. While our findings clearly reveals many converging features between the three NDs, they also reveal some divergence which is to be expected and confirm that the HD, PD and ALS *Drosophila* lines we use each model a unique neurodegenerative disease. We first asked whether the Tip60 HAT/ HDAC2 imbalance we observed during early stages of neurodegeneration in two separate Aβ_42_ and APP *Drosophila* models of AD^17^ is a common deficit in *Drosophila* models in PD, ALS and HD. We show that similar to AD, Tip60 HAT/ HDAC2 neuronal expression balance is disrupted in the disease pathways in all three NDs examined. While the Tip60 HAT expression shows a significant reduction in ALS and a slight but non-specific reduction in PD, it is important to note that there is a significant increase in HDAC2 expression in all three neurodegenerative conditions. Thus, inappropriate HDAC2 upregulation appears to be a common theme in AD, PD, ALS and HD. While decreased Tip60 expression has not previously been reported in ALS, our findings are in general agreement with recent studies that show reduced Tip60 mRNA in a cellular model of PD^50^ and HDAC2 upregulation in both PD^51^ and ALS^15^. Interestingly, we found that both Tip60 and HDAC2 expression levels were upregulated in the *Drosophila* HD model, suggesting a possible compensatory mechanism and that disruption of other HATs may be involved in HD neurodegenerative progression. Another non-mutually exclusive possibility is that the polyglutamate repeat region may bind and sequester Tip60 thereby inactivating it, similar to what has been observed in HD for HATs CBP and PCAF^11^. Thus, the epigenetic mechanistic modes of histone acetylation inactivation in HD may differ somewhat from AD, ALS and PD. Together, our studies reveal that disruption of Tip60 HAT/ HDAC2 homeostasis is a common epigenetic feature conserved in molecularly and clinically distinct NDs.

Epigenetic mediated transcriptional dysregulation of neuroplasticity genes in the brain is a well-characterized key step in many NDs However, many studies characterize such alterations in either aged ND animal models or post-mortem human brains^4,5^, and thus whether such alterations are a cause or a consequence of neurodegeneration in different NDs remains unclear^4,7^. We previously identified a set of critical neuroplasticity genes that are inappropriately repressed during early stages of neurodegeneration progression in the brains of two distinct yet synergistic APP and Aβ_42_ AD *Drosophila* well before adult amyloid plaque formation and lethality occur. These studies also revealed concomitant disruption of HDAC/Tip60 balance with inappropriate enrichment of HDAC2 binding at certain repressed Tip60 neuroplasticity target genes that could be alleviated by increasing Tip60 levels. Here we show that the same early AD associated repressed genes we previously identified (*futsch*, *dlg, sh, dsh*) are also inappropriately repressed during early stages of neurodegenerative progression in the HD, ALS and PD models examined with *futsch* and *dsh* gene repression common in all three diseases. Interestingly, we also observed that *sh* is reduced in Htt128 OE, but not in SNCA or Vap-33 OE models while *dlg* is altered in SNCA, but not in Htt128 OE or Vap-33 OE, suggesting that sh reduction may be linked to HD, whereas dlg reduction may be linked to PD. Similar to AD, each of the repressed Tip60 target neuroplasticity genes (*futsch*, *dlg, sh, dsh*) showed reduced Tip60 binding as well as loss of acetylation at cognition linked Tip60 mediated H4K12 and H4K16 acetylation marks in comparison to healthy wild-type flies of identical developmental staging. These findings suggest that disruption of Tip60 HAT function in epigenetic gene control is a common early event in multiple NDs. Our previous studies and others^52^ demonstrate significant inappropriate upregulation of HDAC2 neural transcripts and recruitment to synaptic plasticity gene loci in both AD fly and mouse models, supporting an HDAC2 specific mechanism underling disease associated gene repression. Similar to AD, here we show inappropriate upregulation of neural HDAC2 transcripts in HD, PD and ALS and a significant increase in inappropriate HDAC2 enrichment at *sh* in both ALS and HD brains and *dlg* and *dsh* in HD larval brains. However, unlike AD, we fail to observe HDAC2 enrichment at the remainder of plasticity gene loci tested. These results suggest HDAC2 independent modes of repression for certain synaptic plasticity genes in NDs and support the need for high resolution genome-wide ChIP-Seq mapping to identify the full repertoire of potential inappropriately regulated HDAC2 gene loci common in different NDs.

Strikingly, the two Tip60 mediated epigenetically misregulated genes (*futsch* and *dsh*) common to HD, PD, ALS and AD play critical roles in regulating synaptic plasticity and long-term potentiation (LTP). For example, microtubule associated protein, *futsch*, binds to atypical protein kinase (aPKC) for regulation of long-term potentiation (LTP) and memory maintenance^53^. Moreover, *dsh* is a downstream effector of the Wnt signaling, which has been shown to facilitate LTP expression^54^. Consistent with these functions, and in general agreement with prior reports^39^, here we observed functional deficits in locomotor ability in all three ALS, PD and HD ND models examined and concomitant alterations in NMJ synaptic morphology in ALS and PD. Although no morphological defects in the NMJ were observed in our HD model, previous literature shows Htt(128Q) expression has functional consequences on synaptic transmission, such as reduction in evoked transmission and quantal size^55^. Further, we observed short term memory deficits in both the HD and PD models with the HD fly model also exhibiting defects in olfactory learning and the ALS fly model exhibiting a non-significant yet downward trend in STM. Thus, early alterations in Tip60 epigenetic mediated control of target neuroplasticity genes may cause the subtle synaptic defects and mild cognitive impairment (MCI) believed to represent a transitional period before full pathophysiology of these NDs appears^56,57^. Our results are in general agreement with recent findings that report early alterations in epigenetic gene control in an HD and AD^17,58–61^, and modest but still early transcriptome changes in ALS and PD^62–64^. We propose that epigenetic Tip60/HDAC2 imbalance occurs early in multiple NDs and triggers certain critical synaptic gene expression alterations that persist and/or initiate additional gene disruptions during later stages of the disease that negatively impact synaptic plasticity and LTP.

**Figure 7:**
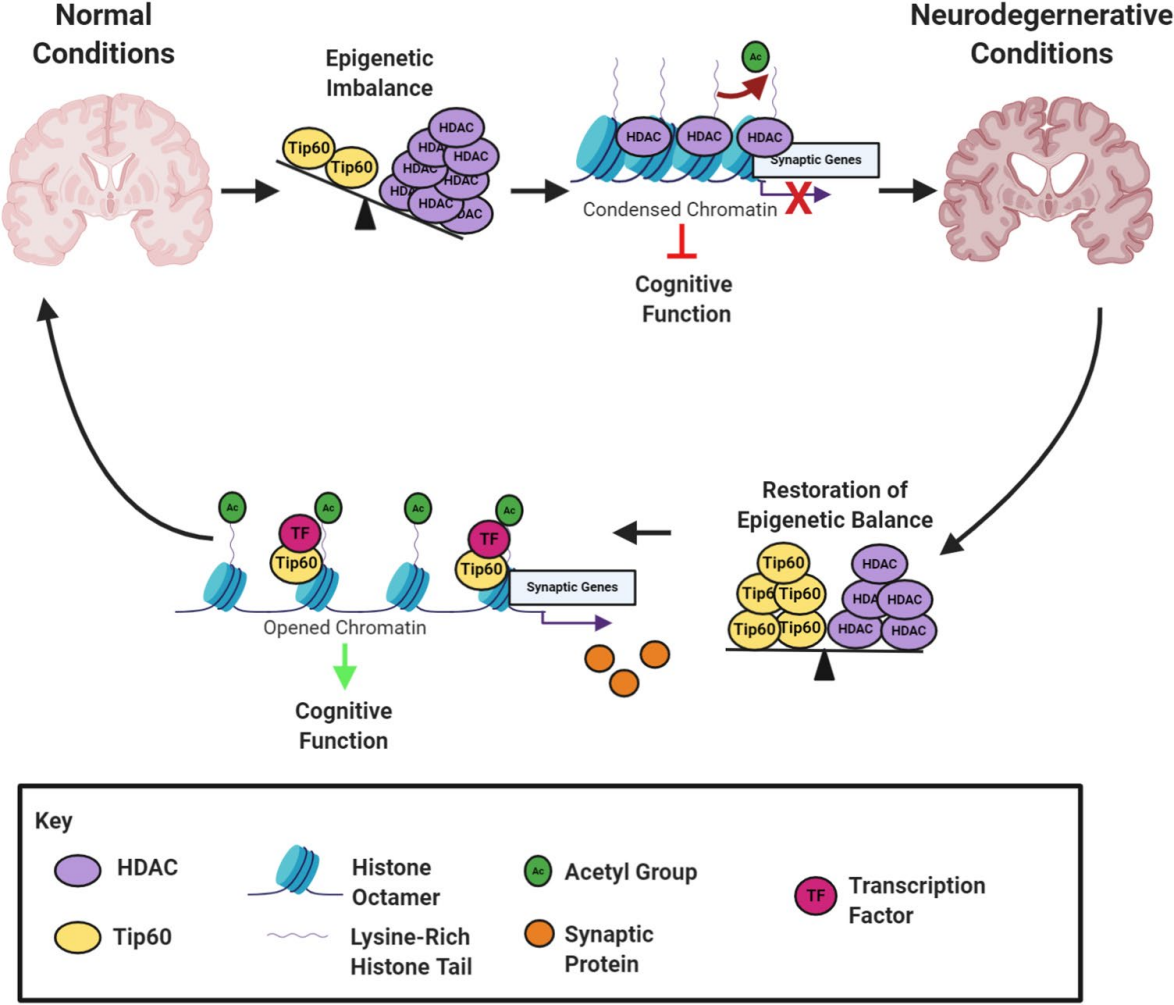
Tip60 promotes neuronal health by protecting against early Tip60 HAT/HDAC imbalance in multiple neurodegenerative diseases. Our results support a model for synaptic defects and cognitive decline in preclinical stages of multiple NDs due to early disruption of Tip60/HDAC homeostasis. This imbalance results in reduction of Tip60 mediated histone acetylation at synaptic gene loci with concomitant gene repression. Increasing cellular levels of specific neural HATs such as Tip60 protects against altered acetylation homeostasis in the brain to maintain appropriate neuroplasticity gene expression profiles and neural health.

We previously demonstrated that increasing Tip60 levels in the AD larval brain restores Tip60 HAT/HDAC2 homeostatic balance at least in part, by decreasing HDAC2 levels. Consequently, we observed Tip60 mediated protection against alterations in neuroepigenetic acetylation signatures, repression of neuroplasticity gene expression profiles and functional deficits in brain morphology and cognition. Here, we show that similar to AD, increased Tip60 levels in the fly brain protects against locomotion deficits in all three HD, PD and ALS ND models tested. In addition, increasing Tip60 levels in the brain protected against the significant STM defects observed in the PD and non-significant deficit trend in the ALS and HD larvae. Our results that Tip60 not significantly rescue STM in the HD model is consistent with our observation that Tip60 was not reduced in the HD larval brain. Together, our results reveal that functional neural deficits common in *Drosophila* models of multiple NDs are alleviated by increasing Tip60 HAT levels in the brain, strengthening the concept that fine-tuning of HAT/HDAC balance is critical for neural function (Fig. 7).

During the last decade, there has been extensive research focusing on restoration of histone acetylation homeostasis in several animal models of NDs. As such, a number of HDAC inhibitors (HDACi) have risen as a promising new strategy for treatment of neurodegenerative conditions that include cognitive deficits, motor impairments and other neuropathological phenotypes^65–69^. However, many HDACi are non-specific and act by increasing global acetylation levels, resulting in unwanted side effects that raise concerns about their therapeutic applicability^7,70–72^. Here, we show that early disruption of HAT/HDAC2 balance is a common feature in multiple NDs, supporting the concept of targeting HAT activity in a combinatorial HAT/HDAC therapeutic approach. To this end, we previously demonstrated that HDACi treatments are not sufficient for full rescue of certain neural gene expression patterns, as they may still require recruitment of Tip60 for site-specific acetylation and/or for interaction with additional transcriptional factors^44^. Further, many HATs have non-redundant functions and studies have shown success in restoring histone acetylation homeostasis in the brain and concomitant neurodegenerative phenotypes by increasing specific HAT levels^7,17,73,74^. For example, overexpression of p300 HAT, but not HDAC inhibition, restored acetylation levels of H3 histone and p53, aiding axonal regeneration in mature retinal ganglion cells after optic nerve injury^75^. Accordingly, HAT activators have emerged as novel potential alternatives to HDACi for neuroprotection^76,77^. In support of this concept, here we show protection against nervous system related locomotion deficits and short term memory loss in multiple ND *Drosophila* models solely by increasing Tip60 HAT levels specifically in the brain. Moreover, our observations that Tip60 HAT/HDAC2 imbalance and concomitant transcriptional dysregulation occur early during neurodegenerative progression, such HAT based therapeutics may be effective for early and general neurodegenerative disease protection. Our findings lay the groundwork for discovering a potentially broad neuroprotective role for Tip60 HAT action in multiple neurological disorders, providing opportunity for future therapeutic intervention.

## Materials and methods

### Fly strains and crosses

All fly lines were raised under standard conditions at 22 °C on standard yeasted *Drosophila* media (Applied Scientific Jazz Mix Drosophila Food, Thermo Fischer Scientific). The pan-neuronal driver *elav*^*C155*^, MB-specific driver *201Y*, *UAS-Htt(128Q), UAS-Hsap/SNCA*^*A30*P^ and *UAS-Vap-33-1*, *Cyo/Sco*, and *TM3/TM6* were all obtained from Bloomington Drosophila Stock Center (Bloomington, IN). For controls for all experiments, the respective UAS or GAL4 driver lines were used alone and yielded identical phenotypes. All experimental crosses were carried out at normal physiological temperature of 25 °C with 12 h light/dark cycle. Controls for all experiments.

### Generation of 201Y; Tip60 fly strain

Generation of the double transgenic *201Y-Gal4*; *UAS-Tip60* line expressing Tip60^WT^ in the mushroom body of the brain followed standard genetic techniques. Balancers with visible phenotypes on the 2nd chromosome (*Cyo/Sco*) and 3rd chromosome (*TM3/TM6*), as well as eye phenotype associated with mini white gene in *201Y-Gal4* and *UAS-Tip60* insertions were used to select for desired genotypes. Briefly, flies containing homozygous *201Y-Gal4 (201Y)* on 2nd chromosome were crossed with *TM3/TM6* whereas flies containing homozygous *UAS-Tip60 (Tip60)* on 3rd chromosome were crossed with *Cyo/Sco.* These crosses resulted in the F1 progeny being heterozygous for the balancer as well as *201Y* or *Tip60*. These flies were then crossed with their balancer counterpart (i.e. *TM3* with *TM6* and *Cyo* with *Sco*), and F2 progeny were selected for *201Y/201Y*; TM3/TM6 or *CyO/Sco*; *Tip60/Tip60*. Next, F2 progeny flies were crossed together, and flies were selected for *201Y*/*CyO*; *Tip60/*TM3 in the next generation. A final self-cross was executed and flies homozygous for *201Y* and *Tip60* (that is, 201Y; Tip60) were selected for experiments. Tip60 overexpression in the larval brains was confirmed by RT-qPCR using a mixture of exogenous and endogenous Tip60 primers. Flies containing an extra ectopic copy of UAS-GFP (*201Y-Gal4; UAS-GFP)* which is the same genetic background as the *201Y-Gal4; UAS-Tip60* used to test Tip60 rescue, showed no significant difference in line-crossing then third instar larvae expressing either Htt(128Q), SNCA^A30P^ or Vap-33-1 under the control of mushroom body (MB)-specific 201Y-Gal4 driver alone. These results confirmed that the Tip60 mediated rescue we observed is not due to GAL4 dilution caused by the extra Tip60 construct.

### Immunohistochemistry

Antibody staining and imaging was performed as previously described^78^. *UAS-Htt (128Q), UAS-Hsap/SNCA*^*A30P*^ and *UAS-Vap-33-1* were crossed with *elav*^*C155*^*-Gal4* for expression of the respective transgene in all neurons. *elav*^*C155*^*-Gal4* crossed to *w*^*1118*^ served as wild type control. Briefly, wandering third instar larvae were dissected in calcium-free Haemolymph-like saline (HL3) saline on a sylgard dish. Fillet preparations were fixed in 4% paraformaldehyde for 30 min, followed by 2 washes in 1X PBS and one with 1 × PBS with 0.2% Triton (PBS-T). Samples were blocked with 5% goat serum for 30 min and then incubated overnight in conjugated primary antibodies at 4 °C. After 2 washes in 1X PBS-T and one wash in 1X PBS, samples were mounted on Vectashield mounting media (Vector laboratories, Burlingame CA). Slides were imaged within 24 h of mounting. Goat anti-HRP primary antibody conjugated with Alexa488 was obtained from Jackson Immunoresearch Laboratories Inc. and used at a dilution of 1:25. Rhodamine phalloidin was obtained from Molecular Probes, Invitrogen and used at a dilution of 1:40.

### Chromatin immunoprecipitation (ChIP)

Chromatin was extracted and sheared from ~ 200third instar larval heads per experiment. To obtain larval heads, the first 1/3 of the larvae (anterior head region) was isolated. Remaining fat bodies were carefully dissected and discarded. All larval heads were inspected visually to ensure that the entire CNS was intact. Because we use a GAL4-inducible system to target gene expression exclusively in the nervous system of the larvae, this method ensures virtually no variability in gene expression in the samples used. For ChIPs, we used truChIP Chromatin Shearing Kit from Covaris following the manufacturer’s instructions. Briefly, protein–DNA cross-links were made at RT for 5 min with 1% formaldehyde and tissue was pulverized using the CryoPrep from Covaris. Cells were lysed and nuclei were prepared using Covaris lysis buffer. Sonication of DNA was performed using a Covaris E220 Ultrasonicator for 13 min. The sheared chromatin was immunoprecipitated using the EZ-Magna ChIP A Chromatin Immunoprecipitation Kit (Millipore) following the manufacturer's instructions. Briefly, ChIP was performed with 30 μg of sheared chromatin using anti-Rpd3 (Abcam, ab1767), anti-Tip60 (Abcam, ab23886), anti-H4K5ac (Active Motif, 39699), anti-H4K12ac (Active Motif, 39166), anti-H4K16ac (Active Motif, 39168), and Normal Mouse IgG Polyclonal Antibody control (Millipore) antibodies. The eluted material from the immunoprecipitation was then purified using a QIAquick PCR purification kit (QIAGEN) and used directly for qPCR. Primer sets were designed by NCBI/Primer-BLAST (www.ncbi.nlm.nih.gov/tools/primer-blast/). Fold enrichment for all the respective genes was calculated relative to the non-specific Mouse IgG Polyclonal Antibody control.

### Imaging and analysis

Larval NMJs were imaged using Olympus FV1000 Fluoview laser scanning confocal microscope. Muscle segmentation was used to locate the NMJ synapses present exactly between muscles 6/7 in the segment A3 of larval body wall. All images were captured using constant confocal gain settings. NMJs were optically sectioned with the help of Olympus FV1000 software and images were acquired as a Z-stack. Later, a 2D projection at maximum intensity was rendered with the help of ImageJ software (NIH). Quantitative analysis of boutons per NMJ, area of NMJ and area of muscle was performed using ImageJ. In order to minimize variation across larval dissection preparations between samples, NMJ area was normalized to the area covered by muscles 6/7 in segment A3 for each sample.

### Larval line crossing assay

Larval locomotion was analyzed as previously described^79^. The line crossing apparatus consisted of a petri plate containing 1% agar positioned on a 0.2 cm^2^ grid paper. A single third instar larva was placed on the petri plate and allowed to acclimate to the plate for 5 min. After initial acclimation, the number of grid lines passed by the head of the larva in 30 s were recorded. This was performed a total of three times for each larva and averaged. Data from thirty larvae were collected.

### Olfactory learning and memory assay

Third instar larvae were trained and tested for olfactory learning and memory performance following guidelines in Honjo and Furukubo-Tokunaga ^46^. Freshly prepared 2.5% agar plates with 2 ml 1 M SUC or DW (for control) spread over the agar were used to train the third instar larvae. 10 µl of undiluted odor Linalool (LIN) (Nacalai, Tokyo, Japan) was spotted on a filter disk and placed on the lid of the petri dish (Fig. 6A). 50 to 100 larvae were transferred to the plate and kept for 30 min. After training, the larvae were rinsed with distilled water and transferred to the test plate. The test plate contained odor (2.5 µl) on one side and none on the other side. The plate was observed for 3 min and responsive index was determined based on the number of larvae (RI) within the 3 cm semicircular area. RI = (number of larvae in the odor area – number of larvae in the control area)/total number for larvae counted. ∆RI was calculated as the difference in RI of LIN/SUC and control LIN/DW. For memory performance test, the larvae were trained as described above, and kept on 2.5% agar plate for the indicated time until the olfactory test was performed. Naïve olfactory and gustatory response test was performed as described in Honjo and Furukubo-Tokunaga^46^. To determine the speed of the larvae from different genotypes, third instar larvae were placed on 2.5% agar plates, allowed to crawl for one minute and video was recorded using a Sony DCR-SR47 Handycam with Carl Zeiss optics. The recorded path length was quantified, and locomotion speed was calculated using the software Tracker. RIs of all the genotype were normalized using their respective locomotion speed.

### RT-qPCR analysis

Total RNA was isolated from 40 staged third instar larval brains using the RNeasy Plus Mini Kit (QIAGEN). Complementary DNA (cDNA) was prepared using SuperScript II reverse transcriptase kit (Invitrogen) according to the manufacturer’s instructions with 1 µg total RNA and 0.2 µg/ml random hexamer primers (Roche Applied Science). PCRs were performed in a 20 µl reaction volume containing cDNA, 1 µM Power SYBR Green PCR Master Mix (Applied Biosystems), and 10 µM of both forward and reverse primers. Primer sequences are available in the Supplementary Table 2. The fold change for each dataset is calculated using the standard ∆∆Ct method using the reference *Drosophila* RP49 gene as the internal control. For the heterochromatin region, 4 µl of 2.5 µM primer set (Active Motif, 71,028) was added to 20ul reaction volume. PCR was performed using an ABI 7500 Real-Time PCR system (Applied Biosystems) following the manufacturer’s instructions. Fold change in mRNA expression was determined by the ∆∆Ct method.

## Supplementary information

Supplementary Information.

## Data Availability

All data generated or analysed during this study are included in this published article (and its Supplementary Information files).
