# Supplementary material for: Disruption of Tip60 HAT mediated neural histone acetylation homeostasis is an early common event in neurodegenerative diseases
Source: Sci Rep. 2020 Oct 26;10:18265. doi: 10.1038/s41598-020-75035-3 (PMC7588445; doi:10.1038/s41598-020-75035-3)
Supplement: Supplementary file 1 — Supplementary Information. [file 41598_2020_75035_MOESM1_ESM.docx]

Disruption of Tip60 HAT mediated neural histone acetylation homeostasis is an early common event in neurodegenerative diseases

Mariah Beaver^1,+^ and Akanksha Bhatnagar^1,+^, Priyalakshmi Panikker^1,¶^, Haolin Zhang^1,¶^, Renee Snook^1^, Visha Parmar^1^, Gayathri Vijayakumar^1^, Nitheesha Betini^1^, Sunya Akhter^1^ and Felice Elefant^1,*^

**Supplementary Data**

| Genotype | Olfactory response (LIN) | Gustatory response (1M SUC) |
| --- | --- | --- |
| Control | 0.35 ± 0.08 | 0.67 ± 0.17 |
| Tip60 | 0.23 ± 0.09 | 0.55 ± 0.06 |
| Htt(128Q) | 0.27 ± 0.18 | 0.53 ± 0.05 |
| Htt(128Q)/Tip60 | 0.41 ± 0.15 | 0.58 ± 0.02 |
| SNCAA30P | 0.2 ± 0.09 | 0.57 ± 0.27 |
| SNCAA30P/Tip60 | 0.26 ± 0.07 | 0.59 ± 0.03 |
| V ap-33-1 | 0.30 ± 0.09 | 0.60 ± 0.03 |
| Vap-33-1/Tip60 | 0.37 ± 0.12 | 0.52 ± 0.09 |

**Supplementary Table 1: Sensory acuities.** Olfactory and gustatory responses were examined as described in materials and methods. Data was obtained from at least 4 independent experiments (n=50/experiment). No significant difference between olfactory or gustatory response was observed between all genotypes. Errors represent SEM.

| Tip60 | CTCGGCCTGAGGCTTGTAAC | CCTTCCACGACCTGAACTCC |
| --- | --- | --- |
| HDAC2 | CGGCAAGTGCGTGGAGTTCG | CTCATAGGTCCAGCAGCGGG |
| sh | GGTGGCATGGCCGCCGTTGC | CTCGAGCTGCTCCTTCTGGTG |
| futsch | CCGCGGCTGAGCAATCCGCCC | GGCGCTTCAGTTTTCCCGGCGCA |
| dlg | TATTTGCCACCGAACAGGCGTTG | ACCCAAATGGTTGGTCCCGACT |
| dsh | ACCCAGCCGCAACGAGTCAA | AACCGATTGCTGCCGGACAC |

**Supplementary Table 2: Primer sequences for RT-qPCR.** These forward and reverse primer sequences were used for mRNA quantification of the respective genes using RT-qPCR as

described in the methods section.

| Disease Phenotype | Huntington’s Disease  Htt(128Q) | Parkinson’s Disease  SNCA^A30P^ | Amyotrophic Lateral Sclerosis (ALS)  VAP-33-1 |
| --- | --- | --- | --- |
| Synaptic plasticity gene expression (RT-qPCR) | ↓ sh, ↓ futsch,  ↓ dsh | ↓ futsch, ↓ dlg,  ↓ dsh | ↓ futsch, ↓ dsh |
| Tip60 HAT/HDAC balance (RT-qPCR) | ↑Tip60, ↑ HDAC2 | ↑ HDAC2 | ↓ Tip60, ↑ HDAC2 |
| Tip60 enrichment  (ChIP-qPCR) | ↓ futsch, ↓ dlg,  ↓ dsh | ↓ sh, ↓ futsch,  ↓ dlg, ↓ dsh | ↓ sh, ↓ futsch,  ↓ dlg, ↓ dsh |
| HDAC2 enrichment  (ChIP-qPCR) | ↑ dlg, ↑ dsh | ↑ sh | ↑ sh |
| H4K16 enrichment  (ChIP-qPCR) | ↓sh, ↓ futsch,  ↓ dlg, ↓ dsh | ↓sh, ↓ futsch,  ↓ dlg, ↓ dsh | ↓sh, ↓ futsch,  ↓ dlg, ↓ dsh |
| H4K12 enrichment  (ChIP-qPCR) | ↓ futsch, ↓ dsh | ↓ futsch, ↓ dsh | ↓ futsch, ↓ dlg,  ↓ dsh |
| Synaptic morphology abnormalities (immunohistochemistry) | -- | ↑ Satellite boutons  ↓ NMJ Area/ muscle area | ↑ Total boutons,  ↑ Type Ib boutons, ↑Type Is boutons, ↑ Satellite boutons |
| Larval locomotion  (line crossing assay) | ↓ Locomotion, rescued by ↑Tip60 | ↓ Locomotion,  rescued by ↑Tip60 | ↓ Locomotion,  rescued by ↑Tip60 |
| Larval olfactory learning assay | ↓ Learning | -- | -- |
| Larval memory assay | ↓ Short term memory | ↓ Short term memory, rescued by ↑Tip60 | -- |

**Supplemental Table 3.**  **Comparison summary of epigenetic, morphological and behavioral phenotypes for three disease models: Huntington’s Disease (Htt(128Q)), Parkinson’s Disease (SNCA^A30P^) and Amyotrophic Lateral Sclerosis (VAP-33-1).** Only statistically significant results are included. Abbreviations: shaker (sh), Discs large 5 (dlg), dishevelled (dsh), neuromuscular junction (NMJ).
